# Occupational exposure to pesticides dysregulates systemic Th1/Th2/Th17 cytokines and correlates with poor clinical outcomes in breast cancer patients

**DOI:** 10.3389/fimmu.2023.1281056

**Published:** 2023-10-24

**Authors:** Stephany Bonin Godinho dos Santos, Janaína Carla da Silva, Hellen dos Santos Jaques, Marina Ferronato Dalla Vecchia, Mariane Okamoto Ferreira, Daniel Rech, Matheus Ryan Noah Sierota da Silva, Roberta Bonin Godinho dos Santos, Carolina Panis, Dalila Moter Benvegnú

**Affiliations:** ^1^ Programa de Pós-Graduação em Ciências Aplicadas à Saúde, Universidade Estadual do Oeste do Paraná, Francisco Beltrão, Paraná, Brazil; ^2^ Department of Biochemistry and Molecular Medicine, Université de Montréal, Montreal, Quebec, Canada; ^3^ Laboratório de Biologia de Tumores, Universidade Estadual do Oeste do Paraná, Francisco Beltrão, Paraná, Brazil; ^4^ Department of Surgery, Hospital de Câncer de Francisco Beltrão, Francisco Beltrão, Paraná, Brazil; ^5^ Curso de Graduação em Engenharia Ambiental e Sanitária, Universidade Tecnológica Federal do Paraná, Francisco Beltrão, Paraná, Brazil; ^6^ Curso de Graduação em Farmácia, União de Ensino do Sudoeste do Paraná, Francisco Beltrão, Paraná, Brazil; ^7^ Programa de Pós-graduação em Saúde, Bem-estar e Produção Animal Sustentável na Fronteira Sul, Universidade Federal da Fronteira Sul, Paraná, Brazil

**Keywords:** breast cancer, prognosis, immune response, cytokines, dysregulation

## Abstract

Pesticides are compounds known to cause immunetoxicity in exposed individuals, which have a potential to substantially modify the prognosis of pathologies dependent on an efficient immune response, such as breast cancer. In this context, we examined the circulating cytokine profile of Th1/Th2/Th17 patterns in women occupationally exposed to pesticides and their correlation with worse prognostic outcomes. Peripheral blood samples were collected from 187 rural working women with breast cancer, occupationally exposed or not to pesticides, to quantify the levels of cytokines IL-1β, IL-12, IL-4, IL-17-A, and TNF -α. Data on the disease profile and clinical outcomes were collected through medical follow-up. IL-12 was reduced in exposed women with tumors larger than 2 cm and in those with lymph node metastases. Significantly reduced levels of IL-17A were observed in exposed patients with Luminal B subtype tumors, with high ki67 proliferation rates, high histological grade, and positive for the progesterone receptor. Reduced IL-4 was also seen in exposed women with lymph node invasion. Our data show that occupational exposure to pesticides induces significant changes in the levels of cytokines necessary for tumor control and correlates with poor prognosis clinical outcomes in breast cancer.

## Introduction

Breast cancer is a multifactorial disease whose origin is influenced by genetic and environmental risk factors. In recent years, growing evidence has been accumulated regarding pesticide exposure’s impact on cancer risk (1–6), and mechanisms include oxidative stress generation, hormonal disbalance, epigenetic changes, and immunological deregulation, among others (7–10).

The immune response is a critical factor in avoiding breast cancer development. Due to the sustained carcinogenic challenges faced by the human body, cancerous or precancerous cells arise lifelong, and most of them are eliminated by a healthy immune system. However, some cells can escape immunosurveillance and origin cancer mainly due to immune failure during its elimination (11).

After tumor establishment, immune responses can also act by favoring its progression (12). In this context, cytokines play a pivotal role by affecting tumor-promoting processes such as growth, invasion, and metastatic capacity (13, 14), determining disease prognosis. Because of this dual role, it is unclear when or why the immune response will work in favor of or against breast cancer. Thus, the exposure of patients carrying breast tumors to pro-carcinogenic factors such as pesticides must negatively affect their immune response and disease evolution.

*In vitro* data have evidenced that pesticide exposure favors malign breast cancer cells’ capabilities as migration, angiogenesis (15) and proliferation (16), which are biological features linked to highly aggressive breast tumors in humans. Also, *in vitro* data has pointed out the immunogenotoxicity of pesticides (16–19). However, little is known regarding the relationship between pesticide exposure, breast cancer behavior and immune response. In the same way, evidence concerning pesticide-induced immune deregulation in breast cancer patients has been recently pointed out, but they are preliminary and allow limited conclusions. It has been reported that rural women occupationally exposed to pesticides have reduced circulating levels of the antitumor cytokines TNF-α and IL-1β (20). In addition, a recent study demonstrated that a specific set of immune response components are affected by occupational exposure to pesticides in breast cancer patients, including tumor CTLA-4 overexpression and systemic IL-12 decrease, specifically in those under intermediate disease risk and recurrence (21).

Considering that breast cancer is a disease with systemic immunological implications (22), such findings suggest that expanding this investigation to more cytokines and other clinicopathological parameters could help establish a systemic cytokine signature linked to disease aggressiveness in women chronically exposed to pesticide mixtures and correlate it to specific prognosis features. To reach this goal, this study characterized the Th1/Th2/Th17 circulating profiles and investigated their relationship to clinicopathological features that are determinants of poor disease prognosis.

## Materials and methods

### Study design

The Institutional Ethics Committee approved this study under CAAE 35524814.4.0000.0107, opinion number 810.501. Only patients who signed the informed consent were included. After screening 422 women, a total of 187 were included. Women attended at the Francisco Beltrão Cancer Hospital – Paraná, Brazil, between 2015 and 2021, from 27 municipalities included in the 8th Health Regional of Paraná, were evaluated. A data collection instrument validated for this population was used to obtain the occupational exposure profile to pesticides (23). The exposure criteria were based on women’s continuous, unprotected, and direct handling of pesticides. Rural women with a history of direct handling of pesticides without wearing protective gloves during the preparation and dilution of the concentrated pesticide solution, or that spray pesticide, and/or were responsible for decontaminating personal protective equipment (PPE), and/or washing of clothes used during spraying, and that reported living at least 50% of their lives under direct pesticide handling at least twice a week during all weeks of the year were classified as occupationally exposed. The unexposed group consisted of urban female workers with no previous or current history of occupational exposure to pesticides (24).

The clinicopathological profile was categorized by collecting data from medical records. The following prognostic information was evaluated, based on the National Comprehensive Cancer Network (NCCN) guidelines (25) and the Saint Gallen Consensus (26): estrogen receptor (ER) and progesterone (PR) expression, human epidermal growth factor receptor 2 (HER2) overexpression, ki67 proliferation index, breast cancer molecular subtype, histological grade, presence of intratumoral emboli, presence of metastases in axillary lymph nodes, presence of distant metastasis, age at diagnosis, menopausal status at diagnosis, body mass index (BMI), the occurrence of recurrence and survival profile in the period studied.

### Sample collection and Th1/Th2/Th17 cytokine profiling

Samples of heparinized peripheral blood were collected and centrifuged for 5 minutes at 4.000 rpm to obtain plasma, which was frozen until the analysis.

To quantify the plasma levels of cytokines, interleukin 1 β (IL-1β), interleukin 12 (IL-12), and tumor necrosis factor-alpha (TNF-α) were measured for the Th1 profile of the patients; interleukin 4 (IL-4) for the Th2 profile and interleukin 17 A (IL-17-A) for the Th17 profile. Enzyme-linked immunoassay commercial kits were used (e-Biosciences^®^, USA). Plasma aliquots were incubated on a plate containing a capture antibody specific for each cytokine, followed by successive washes and incubation with streptavidin-labeled secondary antibody. A specific substrate was added for reaction detection, and the plate read at 642 nm. Results were calculated in pg/ml from standard curve data for each cytokine. The detection limit of the kits was 2 pg/mL.

### Statistical analysis

The statistical study was conducted to assess whether there are differences between the immunological profile of cytokines in cancer patients occupationally exposed and not occupationally exposed to pesticides under different clinicopathological parameters. The sample was also characterized concerning the clinicopathological aspects of the patients, comparing the women exposed and those not occupationally exposed to pesticides.

The frequencies of the categories of each clinicopathological variable were compared for patients belonging to both groups using the chi-square test for adherence. In addition, this same test was used to compare groups according to the categories of each variable. Tests were performed with 5% statistical significance.

The Chi-square test for independence was also performed for each variable to analyze the association between categories and groups. In injury situations with the assumption of a minimum expected frequency of 5, the Monte Carlo method was used as an association test, also with a 5% significance level. The purpose of this test is the same as the Chi-square test for independence. However, it is statistically more robust when the assumptions are not verified. Data analyzes were performed using the R software (27).

For cytokine level analyses, GraphPad Prism version 9.0 was used. Data distribution was tested by using the Shapiro-Wilk test. Variables normally distributed were analyzed with parametric tests, and nonparametric tests were used to analyze the nonparametric ones (Student’s t-test or the Mann-Whitney test, respectively). Data are presented as box-plot and described in results as means (parametric data) or medians (nonparametric data). For all analyses, a p ≤ 0.05 was considered significant. P values are shown in **Table 1** as follows: p-value1 of the chi-square test for adherence comparing the categories of each variable for patients not exposed to pesticides; p-value2 of the chi-square test for adherence comparing the categories of each variable for patients exposed to pesticides; p-value3 of the Chi-square test for adherence comparing the groups according to the categories of each variable. A multivariate analysis, based on the principal component analysis (PCA) was conducted concerning cytokines and pesticide exposure. Only data with a p-value < 0.05 were showed as Figures.

**Table 1:** Frequency (n) and percentage (%) of clinicopathological data (discrete variables) considering exposure to pesticides.

		Not exposed		Exposed		
Variable	Category	%	p-value_1_	%	p-value_2_	p-value_3_
Estrogen receptors	Negative	19.72	**<0.0001**	22.52	**<0.0001**	**0,0127**
	Positive	64.79		62.16		**0,0024**
	NA	15.49		15.32		
Progesterone receptors	Negative	42.25	0.8563	41.44	0.8834	**0,0094**
	Positive	43.66		42.34		**0,0104**
	NA	14.08		16.22		
HER2 expression	Negative	78.87	**<0.0001**	78.38	**<0.0001**	**0,0002**
	Positive	7.04		5.41		0,6698
	NA	14.08		16.22		
Ki67%	14	33.80	**0.0186**	37.84	0.1447	**0,0017**
	14	52.11		46.85		**0,0245**
	NA	14.08		15.32		
Tumor aggressiveness	Less aggressive	36.62	0.1032	40.54	0.6600	**0,0014**
	More aggressive	49.30		43.24		**0,0436**
	NA	14.08		16.22		
Molecular subtypes	Luminal A	29.58	**0.0009**	34.23	**<0.0001**	**0,0017**
	Luminal B	30.99		6.31		0,1617
	HER2	18.31		16.22		0,4142
	Triple-negative	14.08		29.58		0,1336
	NA	34.23		30.99		
Tumor size (mm)	20	32.39	**0.0394**	32.43	**0.0008**	**0,0167**
	20	47.89		53.15		**0,0002**
	NA	19.72		14.41		
Histological grade	1	26.76	0.1433	23.42	**<0.0001**	0,1400
	2	36.62		45.05		**<0,0001**
	3	22.54		15.32		0,8055
	NA	14.08		16.22		
Intratumoral emboli	No	66.20	**<0.0001**	54.05	**<0.0001**	**0,0755**
	Yes	19.72		29.73		**<0,0001**
	NA	14.08		16.22		
Lymph nodal metastasis	None acometed	60.56	**<0.0001**	53.15	**0.0026**	**0,0251**
	At least one acometed	23.94		34.23		**<0,0001**
	NA	15.49		12.61		
Distant metastasis	No	76.06	**<0.0001**	76.58	**<0.0001**	**0,0002**
	Yes	8.45		10.81		**0,0455**
	NA	15.49		12.61		
Diagnosis	Early	40.85	**0.0291**	35.14	**<0.0001**	0,2195
	Late	59.15		64.86		**<0,0001**
Menopause at diagnosis	No	32.39	**<0.0001**	30.63	**<0.0001**	**0,0008**
	Yes	66.20		65.77		**0,0394**
	NA	1.41		3.60		
Trophic-adipose status	Eutrophic	38.03	**0.0247**	32.43	**<0.0001**	0,1088
	Overweight/Obese	56.34		61.26		**0,0001**
	NA	5.63		6.31		
Chemoresistance	No	63.38	**<0.0001**	63.06	**<0.0001**	**0,0010**
	Yes	19.72		19.82		0,0593
	NA	16.90		17.12		
Recurrence	No	83.10	**<0.0001**	86.49	**<0.0001**	**<0,0001**
	Yes	12.68		11.71		0,2278
	NA	4.23		1.80		
Death	No	90.14	**<0.0001**	94.59	**<0.0001**	**<0,0001**
	Yes	9.86		5.41		0,6949

NA, data not available. The frequency of the NA category was not considered in the statistical analyses. Values in bold indicate that there was a statistical difference between the categories of the variable.

## Results

We included 187 women in the study (111 occupationally exposed to pesticides and 71 non-exposed). As shown in **Table 1**, estrogen receptor was positive in about 65% of cases in the unexposed group, while HER2 expression was negative in 78.87% of women in this group. The ki67 was over or equal to 14% for most cases (52.11%). The most frequent molecular subtypes were Luminal A (29.58%) and Luminal B (30.99%). Tumor size was greater than 20 mm (47.89%), with the prevalence of intratumoral emboli (66.20%). No lymph node was affected in 60.56% of the women; distant metastasis was found in 8.45%. In most cases, women were in menopause (66.20%) and overweight/obese (56.34%). Most of the patients were responsible for the first-line treatment (cytotoxic chemotherapy, 63.38%) without disease recurrence (83.10%) or death (90.14%). There was no statistical difference between the categories of variables PR, tumor aggressiveness according to its molecular subtype (more aggressive = triple-negative *vs*. less aggressive = Luminal), and histological grade.

Concerning women exposed to pesticides, it was found that the ER was positive in about 62% of the cases, and the amplification of HER2 was negative in 78.38% of the women in this group. The most common molecular subtypes were Luminal A (34.23%) and triple-negative (29.58%). Tumor size was greater than 20 mm (53.15%), with histological grade 2 (45.05%) and absence of intratumoral emboli (54.05%). No lymph node invasion was found in 53.15% of the women, and distant metastasis was found in 10.81%. In most patients in this group, women were in menopause (65.77%) and overweight/obese (61.26%). Most were responsible for the first-line treatment (63.06%) without recurrence (86.49%) or death (94.59%) in most patients. There was no statistical difference between the categories of variables PR, ki67>14%, and tumor aggressiveness.

In exposed patients, higher lymph nodal invasion was identified (34.23%) compared to non-exposed women (23.94%) (p-value 3 <0.0001; **Table 1**). We also found higher BMI in this group, indicating overweight or obesity (p-value 3 = 0.0001; **Table 1**).

**Figure 1:**
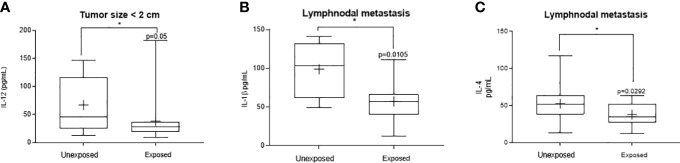
Significant variants in the systemic Thl profile of women with breast cancer occupationally exposed or not to pesticides. The Thl profile was determined through plasma levels of interleukin 12 (IL-12), interleukin 1β (IL-1β,) and tumor necrosis factor alpha (TNF-α). Levels detected in the exposed and non-exposed groups according to the following clinicopathological variables: **(A)** – Tumorsize under 2 cm for IL-12, **(B)** – presence of lymph node metastasis for IL-1β and **(C)** – Lymphnodal metastasis for IL-4. Data are shown as a box-plot of minimum, maximum and median variations. + represents the mean of each group. The p values are shown in the graphs, p<0.05 was considered significant.

**Figure 1** shows the significant changes observed concerning cytokines from the Th1 axis. Reduced IL-12 was observed in exposed patients carrying tumors lower than 2 cm (**Figure 1A**, range: 12.70-147.1 pg/mL to unexposed and 9.29-182.8 pg/mL to the exposed ones, p = 0.051). Patients with lymph nodal invasion presented a reduced IL-1β (**Figure 1B**, range: 48.80-141.2 pg/mL to unexposed and 12.70-111.2 pg/mL to the exposed ones, p = 0.0105). Regarding IL-4 levels, a significant reduction was observed in the group of exposed women with lymph nodal invasion (**Figure 1C**, range:13.07-117.1 pg/mL to unexposed and 12.00-63.01 pg/mL to the exposed ones, p = 0.0414).

**Figure 2:**
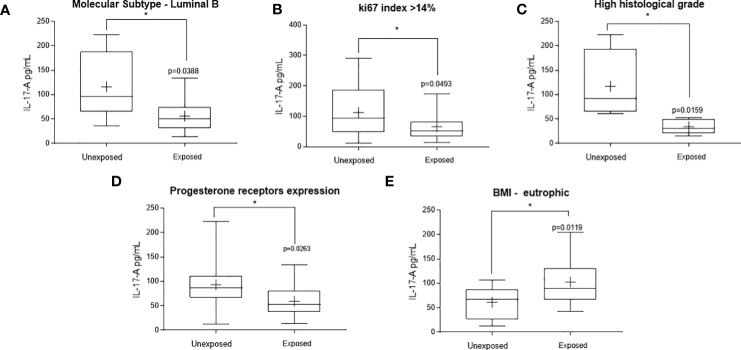
Significant variations in the systemic Thl7 profile of women with breast cancer occupationally exposed or not pesticides. The Thl7 profile was determined by measuring plasma interleukin 17 A (IL-17A). Levels detected in the exposed and non-exposed groups according to the following clinicopathological variables: **(A)** – Luminal molecular subtype B, **(B)** – ki67 proliferation index, **(C)** – tumor histological grade, **(D)** – Presence of progesterone receptors and **(E)** – Eutrophic patients. Data are shown as a box-plot of minimum, maximum and median variations. + represents the mean of each group. The p values are shown in the graphs, p<0.05 was considered significant.

Main variations were observed in the Th17 axis, represented here by IL-17-A (**Figure 2**). A significant decrease in circulating levels of this cytokine was observed in patients occupationally exposed to pesticides, when compared to those not exposed, in the following conditions: carriers of luminal molecular subtype B tumors (**Figure 2A**, range: 36.36-222.7 pg/dL to the unexposed and 13.52-133.7 pg/dL to the exposed ones, p = 0.0176), with high proliferation tumors (ki67 >14%, **Figure 2B**, range: 12.12-291.1 pg/mL to the unexposed and 13.52-173.00 pg/mL to the exposed ones, p = 0.0493), with high histological grade tumors (**Figure 2C**, range: 60.80-222.70 pg/mL to the unexposed and 15.02-52.88 pg/mL to the exposed ones, p = 0.0159) and in those with progesterone receptor-positive tumors (**Figure 2D**, range: 12.12-221.70 pg/mL to the unexposed and 13.52-133.7 pg/mL to the exposed ones, p = 0.0263). A significant increase in IL-17-A was observed in eutrophic patients exposed to pesticides compared to those not exposed (**Figure 2E**, range: 12.12-106.7 pg/mL to the unexposed and 42.52-204.1 pg/mL to the exposed ones, p = 0.0119).

**Figure 3:**
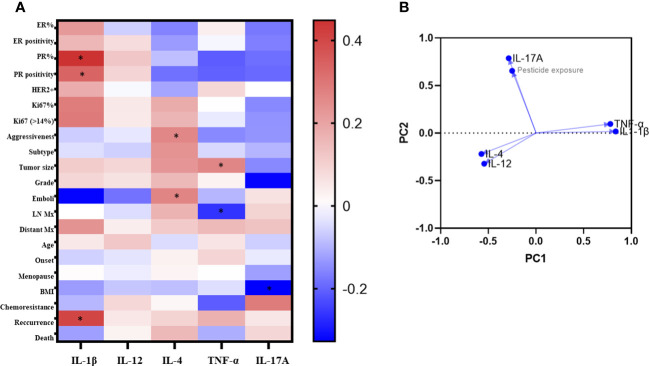
Correlaion analysis of clinicopathological data according to systemic levels of cytokines of exposed breast cancer patients. In **A**, the heatmap of Spearman's R values. Red squares indicate positive correlations. Blue squares indicate the negative ones. As intense the color as stronger the correlation (range from 0 = no correlation to 1 = total correlation). *p<0.05. In **B**, the principal component analysis. ER, estrogen receptors; PR, Progesterone receptors; HER2, amplification to the receptor of the human epidermal growth factor 2; LN, lymphnodal; Mx, metastasis.

Spearman’s correlation analysis (**Figure 3A**) performed in the exposed patients’ group showed that PR positively correlated to IL-1β levels (R = 0.4481 for percent expression and 0.3373 for the presence of PR, p<0.05). For IL-4, positive correlations were found between its levels and disease aggressiveness (R = 0.2613, p<0.05), as well as the presence of intratumoral emboli (R =0.2678, p<0.05). TNF-α levels positively correlated to tumor size (R = 0.2624, p<0.05) and negatively to lymph nodal invasion (R = -0.2633, p<0.05). For IL-17-A, a negative correlation was found concerning BMI categorization (R = - 0.3276, p< 0.05). No significant correlations were observed regarding IL-12.

**Figure 3B** shows the results from PCA analysis among cytokine levels and pesticide exposure. The principal component 1 (PC1) strongly correlated positively to IL-1β and TNF-α (loadings 0.836 and 0.779, respectively), while the principal component 2 (PC2) strongly correlated positively to pesticide exposure and IL-17-A (loadings 0.654 and 0.786, respectively).

## Discussion

Immune response polarization is crucial to determine the outcome of diseases whose prognosis depends on this, such as breast cancer. In this study, we demonstrated that chronic and continued exposure to pesticides significantly and simultaneously affects the levels of circulating Th1/Th2/Th17 cytokines in association with clinicopathological characteristics of worse prognosis.

Pesticides are immunotoxic by multiple mechanisms, interfering with innate and adaptative responses that are crucial against cancer (8), and it is suggested that the chronic antigenic stimulus due to continuous pesticide exposure can induce immune exhaustion (10). In this context, the imbalance in the production of cytokines enrolled in carcinogenesis is described (28).

The immune response against cancer has as its central mechanism a network of cytokines, whose production and signalling work in a homeostatic way within the T helper polarization patterns to effectively combat tumours (29, 30). The Th1 response, represented in our work by the circulating levels of IL-1β, IL-12, and TNF-α, was negatively affected by pesticide exposure, resulting in more aggressive clinicopathological conditions. For example, we observed depletion of IL-12, a main tumor-fighting cytokine. Specifically, women with tumors smaller than 2 cm exposed to pesticides had lower circulating levels of IL-12 compared to non-exposed women. This cytokine has potent antineoplastic activity by inducing a Th1-type response and tumor rejection (31), correlated with increased survival (32). Failure to produce it, even at a stage where the tumor represents a small mass, can influence the development of large tumor masses in the long term, potentially resulting in aggressive tumor behaviours such as the occurrence of metastases observed in such exposed patients (33).

In the present study, we observed that IL-1β levels were reduced in patients with lymph node metastasis, reinforcing the immune dysfunction reported in breast cancer patients exposed to pesticides reported by others (34–36). Th1-mediated immunity is known for its antitumor activity and is associated with longer life expectancy, unlike patients with tumors associated with Th2 subpopulation markers, with a more unfavorable prognosis (37). Thus, the pesticide-driven reduction of Th1 cytokines observed here may represent a substantial impairment for the immune responses against breast cancer.

Some mechanisms are pointed out concerning how pesticide exposure can affect Th1 cytokines. Our study population is under chronic handling of a mix of glyphosate, atrazine, and 2,4D pesticides. Atrazine, for example, changes the secretome pattern of immunoregulatory compartments as the mesenchymal stromal stem cells, attenuating Th1-related molecule production (38). A study (39) investigating the *in vitro* impact of this mixture at low concentrations demonstrated significant disturbances in macrophage polarization in association with a decrease in pro-inflammatory cytokine secretion. Healthy greenhouse workers occupationally exposed to pesticides exhibit significant reduction of circulating pro-inflammatory cytokines such as IL-2, IL-8, IL-12p70 and IFN-γ (35), and the same depletion pattern has been reported for exposed macrophages (40). These data support the idea that pesticides may have a direct deleterious effect on immune cells, which could explain our findings concerning Th1 cytokines depletion in poor prognosis patients. Human data in this context is scarce, which highlights the relevance of the present investigation.

We further demonstrated that pesticide exposure substantially modified the circulating levels of IL-17-A, a Th17 cytokine. The responses modulated by this axis involve innate and adaptive immunity inflammatory processes, affecting the production of other cytokines that modulate breast cancer progression (41). We observed a significant reduction in IL-17-A levels in patients occupationally exposed to pesticides compared to the non-exposed group under several conditions that determine a worse prognosis, such as in patients with proliferative and high-grade tumors. No data was found in literature about IL-17A deregulation in the context of pesticide exposure and breast cancer. In non-cancer conditions, IL-17A levels do not vary in workers exposed to pesticides (42), but *in vitro* and *in vivo* studies show that IL-17-A is related to cancer mechanisms (41, 43, 44). Distinct pesticides seem to act by different mechanisms on Th17 axis. For example, paraquat enriches the gene expression for IL17 signalling in human cells (45). Murine exposure to glyphosate leads to IL-17A reduction in peripheral blood at low doses and has been linked to immune deregulation across generations (46).

Pesticide exposure also augmented IL-17A in eutrophic breast cancer patients. Despite obesity dysregulates IL17-A production (47), and no data was found concerning its meaning in eutrophic patients, these findings support that pesticide exposure induce significant immunological changes in breast cancer patients, which vary according to the clinicopathological status of patients.

Our findings suggest that the combination of both pesticide exposure and breast cancer depletes this cytokine systemically in exposed women. PCA analysis reinforced the strong correlation between this cytokine and pesticide exposure. No data was found regarding atrazine or 2,4D exposures, and there is no information concerning IL-17A changes in the context of breast cancer and pesticide exposure.

Our study has limitations, including the need for measuring other cytokines, the single-point analysis instead of multiple collection points, and the modest sample size. Despite this, we believe that the main novelty and contribution relies on the fact that this is the first study to point out systemic changes in cytokine profiles induced by human exposure to pesticides in the context of breast cancer. Although the specific mechanisms by which pesticides induce such changes in breast cancer patients are unclear, our data reinforce pesticide exposures as potential immunological disruptors of cytokines produced in the immune response against breast cancer, especially in clinical conditions linked to worse prognoses.

## Data availability statement

The raw data supporting the conclusions of this article will be made available by the authors, without undue reservation.

## Ethics statement

The studies involving humans were approved by State University of West Paraná Ethics Committee. The studies were conducted in accordance with the local legislation and institutional requirements. The participants provided their written informed consent to participate in this study.

## Author contributions

SG: Data curation, Investigation, Writing – original draft. JS: Formal Analysis, Investigation, Writing – original draft. HJ: Data curation, Formal Analysis, Writing – original draft. MD: Data curation, Writing – original draft. MF: Data curation, Methodology, Writing – original draft. DR: Data curation, Investigation, Supervision, Writing – original draft. MS: Investigation, Resources, Writing – original draft. RS: Methodology, Writing – original draft. CP: Conceptualization, Data curation, Formal Analysis, Funding acquisition, Investigation, Methodology, Project administration, Resources, Software, Supervision, Validation, Visualization, Writing – original draft, Writing – review & editing. DB: Supervision, Writing – original draft.
